# Range-Dependent Channel Calibration for High-Resolution Wide-Swath Synthetic Aperture Radar Imagery

**DOI:** 10.3390/s24113278

**Published:** 2024-05-21

**Authors:** Man Zhang, Zhichao Meng, Guanyong Wang, Yonghong Xue

**Affiliations:** 1 School of Electronic and Communication Engineering, Guangzhou University, Guangzhou 510006, China; manzhang401@gzhu.edu.cn; 2 Key Laboratory of On-Chip Communication and Sensor Chip, Guangdong Higher Education Institutes, Guangzhou 510006, China; 3 School of Electronics and Communication Engineering, Sun Yat-sen University, Shenzhen 518107, China; 4 School of Information and Science, North China University of Technology, Beijing 100144, China; guanyongwang@ncut.edu.cn; 5 Beijing Institute of Tracking and Communication Technology, Beijing 100094, China; sanger_xue@126.com

**Keywords:** high-resolution and wide-swath (HRWS), synthetic aperture radar (SAR), range-dependent channel phase error, sharpness of the reconstructed Doppler spectrum

## Abstract

High-resolution and wide-swath (HRWS) synthetic aperture radar (SAR) imaging with azimuth multi-channel always suffers from channel phase and amplitude errors. Compared with spatial-invariant error, the range-dependent channel phase error is intractable due to its spatial dependency characteristic. This paper proposes a novel parameterized channel equalization approach to reconstruct the unambiguous SAR imagery. First, a linear model is established for the range-dependent channel phase error, and the sharpness of the reconstructed Doppler spectrum is used to measure the unambiguity quality. Furthermore, the intrinsic relationship between the channel phase errors and the sharpness is revealed, which allows us to estimate the optimal parameters by maximizing the sharpness of the reconstructed Doppler spectrum. Finally, the results from real-measured data show that the suggested method performs exceptionally for ambiguity suppression in HRWS SAR imaging.

## 1. Introduction

The utilization of synthetic aperture radar (SAR) techniques necessitates the production of imagery that possesses the potential to simultaneously achieve high resolution and wide-swath (HRWS) coverage [1,2]. Nevertheless, it is necessary to limit the observed swath of a traditional SAR system in order to obtain clear and precise echoes. This limitation is imposed by the inherent trade-off between Doppler ambiguity and spatial resolution, which is applicable to single-channel radar systems [3]. The digital beamforming (DBF) approaches can help overcome this fundamental limitation by utilizing an azimuth multi-channel system setup [4,5,6] to process the signals received on many channels. The signal received from each channel is recorded and digitized simultaneously. The HRWS SAR imagery can be generated by reconstructing the unambiguous and full Doppler spectra using a Doppler spectra reconstruction with DBF processing. This approach alleviates the contradiction between distance and Doppler ambiguity found in conventional single channel SAR systems, enabling wide-swath and high-resolution imaging. Normally, multi-channel HRWS SAR systems [7,8,9] can be classified into two types—multiple antenna systems and distributed systems. A number of unique algorithms utilizing the DBF technique have been presented in the literature for the purpose of resolving Doppler ambiguity in the spatial domain [10,11,12]. The utilization of azimuth multi-channel SAR with DBF processing is inherently a proficient approach for the development of unambiguous HRWS imagery in practical applications. Nevertheless, the efficacy of ambiguity suppression would be greatly compromised by inevitable channel errors, particularly when monitoring a high contrast scene with prominent targets such as a robust ship under underwater conditions. The calibration of channel mismatch is an essential requirement in order to produce HRWS imagery in azimuth multi-channel SAR systems.

As a result of unforeseen systematic and circumstantial circumstances, such as the incongruity between the characteristics of the transmitting and receiving channels, the presence of channel imbalance introduces additional phase errors among numerous channels. This results in a significant degradation of the performance of DBF Doppler spectra reconstruction. In order to effectively utilize the receiving beamforming approach for Doppler spectrum reconstruction, it is imperative to ensure the proper calibration of the multi-channel SAR system. This calibration process involves adjusting for any phase and amplitude imbalances that may exist across the channels. The occurrence of phase errors during the DBF process can lead to undesirable effects such as spectrum folding and mismatch, which, in turn, result in significant ambiguities in the acquired SAR images. The inclusion of these errors will result in a degradation of the azimuth ambiguity-to-signal ratio (AASR) [13] in the high-resolution wide-swath (HRWS) imagery.

Typically, calibrating approaches are anticipated to effectively mitigate the presence of phase imbalance errors. In recent times, a collection of novel calibration algorithms [14,15,16,17] has been created, which can be broadly categorized into two types—inner calibration and data-driven calibration. The inner calibration approaches involve compensating for azimuth channel imbalance through the incorporation of an additional system. However, the practical implementation of these methods is hindered by the resultant increase in system complexity, hence limiting their applicability in real-world scenarios. For instance, in small-satellite constellation programs, designing extra internal calibration systems for these picosatellites becomes a challenging task, due to restrictions in payload size and power. In contrast, data-driven calibration algorithms aim to predict channel imbalance only from data, employing an adaptive approach that does not introduce additional system complexity. This research focuses solely on the calibration of HRWS SAR imaging using data-driven approaches. Subspace projection methods have been extensively studied in numerous studies within the realm of data-driven calibration algorithms. The channel phase defects for multi-channel SAR high-resolution wide-swath (HRWS) imaging were rectified using a consistent fundamental approach. The estimation of the covariance matrix was conducted in the range-Doppler domain. Subsequently, the covariance matrix underwent eigenvalue decomposition to derive the signal subspace and noise subspace. The subspace containing the signal was formed by the signal steering vectors that had phase errors and these vectors were orthogonal to the subspace containing the noise. Imbalanced phase errors are typically obtained by minimizing the projection of the estimated steering vectors onto the noise subspace. It is commonly considered that the phase faults associated with imbalance are invariant over space. In a previous study [18], a novel auto-calibration approach was introduced based on virtual-source calibration. This algorithm was designed to accurately predict channel errors in a distributed multi-receive radar system. The data from many channels in the azimuth time domain are transformed into the Doppler domain using Fourier transform. Within each Doppler bin, signal elements that correspond to distinct directions are considered as corrective virtual sources. In methods such as subspace projection [19,20], it is anticipated that the iterative optimization process of projecting into the noisy subspaces will yield the element phase errors.

Subspace projection methods are commonly used to calibrate channel phase imbalance in multi-channel HRWS SAR systems. However, subspace projection methods require the spatial degree of freedom to be larger than the ambiguity, which, in turn, requires the use of more channels and complex equipment. The authors of [21,22] present a channel calibration method for HRWS SAR images. This method focuses on addressing phase errors by evaluating the sharpness of the reconstructed Doppler spectrum using a group of ideal spatial filters. The sharpness optimization calibration requires no additional degree of freedom, significantly relaxing the system requirement. In all of the above algorithms, the channel imbalance is assumed to be constant, meaning that it is neither time-variant nor spatially dependent. It is generally acceptable to simplify the time-variant imbalance in a constant model during a system operation period. However, in real HRWS SAR systems [23,24,25], there may be spatially dependent channel imbalance. The antenna pattern can be significantly inconsistent in both phase and amplitude, especially when some channels gradually degrade over time. This imbalance in the antenna pattern will result in the significant spatial dependence of the channel imbalance. The presence of this spatially dependent imbalance limits the current calibration performance of HRWS SAR, which is the reason for our extended work.

In this paper, we developed a range-dependent sharpness optimization-based imbalance correction algorithm for azimuth multi-channel SAR imagery. The approach assumes that the error in the imbalance phase is spatially invariant and can be modeled using a low-order polynomial. The accurate reconstruction of the HRWS complete Doppler spectrum is contingent upon the effectiveness of channel phase correction. The reconstructed Doppler spectra will present energy concentrated into the synthetic antenna pattern format in the Doppler domain, when the range-dependent imbalance phase errors are compensated precisely. Because the existence of the imbalance phase leads to the failure of Doppler ambiguous resolving filtering and the reconstructed Doppler spectra present flattened Doppler formations with low sharpness values. In the range-dependent phase calibration, the estimation of the channel phase errors are transferred into maximizing the sharpness function of the reconstruction of the full Doppler spectra by compensating the range-dependent phase errors within the ambiguity resolving filtering. The range-dependence of the imbalance phase is modeled using a linear polynomial, which is usually precise enough. This algorithm calibrates the range-dependent phase imbalance in a robust and fast manner by jointly using all range bins without blocking processing. Detailed comparative experiments are presented with real SAR data to demonstrate the optimal performance of the proposal.

This paper is organized as follows: In Section 2, the basic mathematic model of the Doppler ambiguity signal with imbalance model and the ambiguity removing filtering is shown. Section 3 encompasses the proposed channel phase calibration. Section 4 assesses the enhanced comparison performance of the suggested approach and draws certain conclusions.

## 2. Basic Mathematic Model Construction

In this section, we mainly construct the basic mathematic model of HRWS SAR imaging, including the azimuth multi-channel SAR geometry and signal models for HRWS imaging, the technique of Doppler ambiguity resolving via inverse-filtering processing, and the range-dependent channel phase error.

### 2.1. Geometry and Signal Models

**Figure 1:**
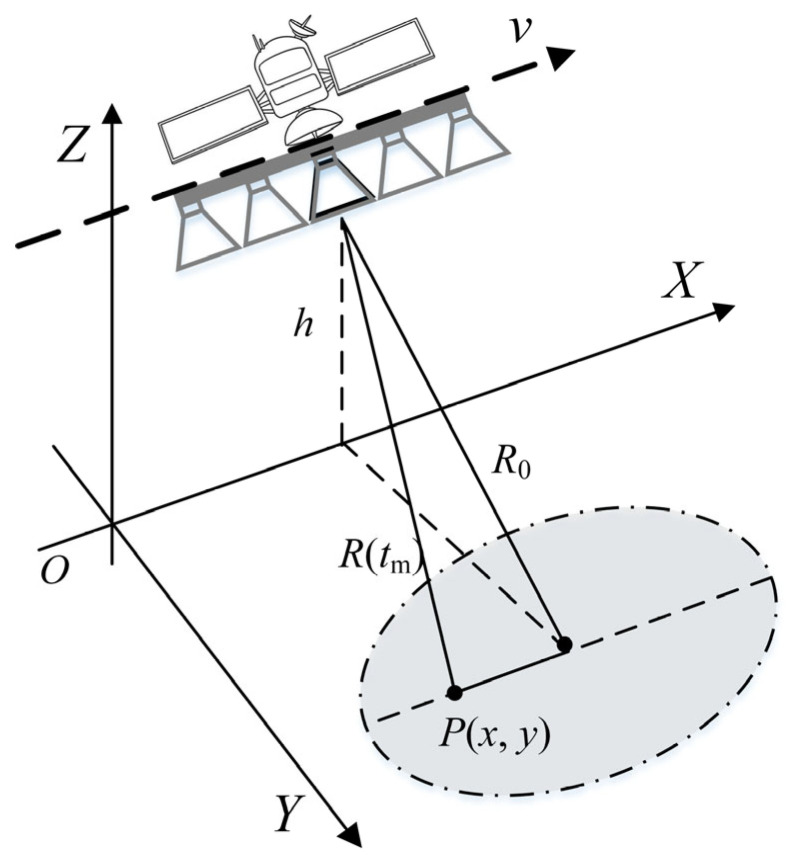
Geometry of HRWS SAR imaging.

Figure 1 depicts the imaging geometry of an azimuth multi-channel SAR system. The platform flies along the x-axis at a constant speed, v, and maintains a fixed altitude, h. It transmits a wide-band signal via the reference channel, and all channels simultaneously receive the echoes. Through phase compensation [9], the echoes generated by a multi-channel SAR system can be made equivalent to those of a single-channel SAR system. It is assumed that a multi-channel HRWS SAR system has M channels. The antenna phase center (APC) of the mth channel is $\left(x_{m},0,h\right)$ at the time t=0 and $\left(x_{m}+vt,0,h\right)$ at time t. The echoes reflected from point $P\left(x,y\right)$ and received by the mth channel at time t are given in [26].

(1) $$s_{m}\left(\tau ,t\right)=\iint \sigma \cdot s\left[\tau -\frac{2r_{m}\left(x,y,t\right)}{c}\right]\cdot w_{a}\left(t-\frac{x-x_{m}}{v}\right)\cdot \exp\left[-j4\pi \frac{r_{m}\left(x,y,t\right)}{\lambda }\right]dxdy$$

where τ and t denote the range fast time (the “range fast time” represents the variable that changes over the duration of the transmitted signal) and azimuth slow time (the “azimuth slow time” refers to the variable that changes among different pulses), respectively. σ is the complex scattering coefficient of a target, and c is the light speed. $w_{a}\left(t-\frac{x-x_{m}}{v}\right)$ is the azimuth window function caused by antenna azimuth pattern and the unnecessary range window function is disregard here. In addition,

(2) $$r_{m}\left(x,y,t\right)=\sqrt{{\left(x-x_{m}-vt\right)}^{2}+R_{b}^{2}}$$

is the instantaneous range between the mth APC and targets and $R_{b}^{2}=\sqrt{y^{2}+h^{2}}$. In addition,

(3) $$s\left(\tau \right)=\mathrm{rect}\left(\frac{\tau }{T_{p}}\right)\cdot \exp\left(j\pi \gamma \tau^{2}\right)$$

is the transmission signal. Where $\mathrm{rect}\left(\cdot \right)$ is the rectangle window function, $T_{p}$ is the pulse duration, and γ is the chirp rate;

By applying a 2D Fourier transform (FT) on (1) and converting it into the two-dimensional (2D) frequency domain, we may derive the following equation utilizing the concept of instantaneous wavenumber:

(4) $$S_{m}\left(f_{r},f_{d}\right)=S_{0}\left(f_{r},f_{d}\right)\cdot \exp\left(-j2\pi \frac{x_{m}}{v}f_{d}\right)$$

where $f_{r}$ is the range frequency and $f_{d}$ is the Doppler frequency. In addition,

(5) $$S_{0}\left(f_{r},f_{d}\right)=\iint \sigma \left(x,y\right)\cdot S\left(f_{r}\right)\cdot W_{a}\left(f_{d}\right)\cdot \exp\left(-jR_{b}\sqrt{{\left(\frac{4\pi f_{r}}{c}\right)}^{2}-{\left(\frac{2\pi f_{d}}{v}\right)}^{2}}\right)\cdot \exp\left(-j2\pi f_{d}\frac{x}{v}\right)dxdy$$

where $S_{m}\left(f_{r},f_{d}\right)$, $S\left(f_{r}\right)$, and $W_{a}\left(f_{d}\right)$ are the frequency spectrums of $s_{m}\left(\tau ,t\right)$, $s\left(\tau \right)$, and $w_{a}\left(t\right)$, respectively.

**Figure 2:**
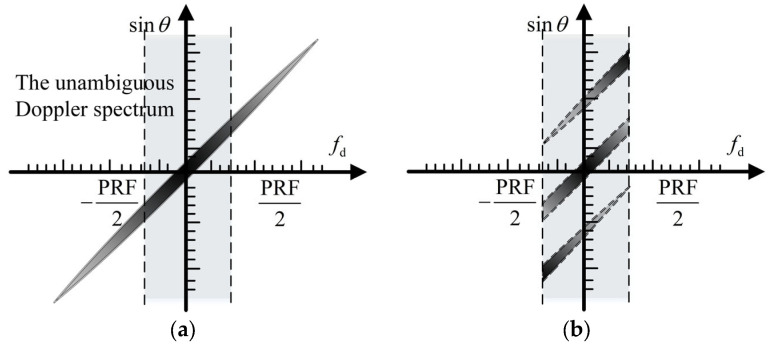
Spatial–time spectrum without and with Doppler ambiguity. (**a**) Without Doppler ambiguity. (**b**) With Doppler ambiguity.

From Equations (4) and (5), one can see that there is only the linear phase difference $\exp\left(-j2\pi \frac{x_{m}}{v}f_{d}\right)$ between the mth and the reference APC for the same Doppler bin, $f_{d}$. In general, Doppler frequency, $f_{d}$, and target angle, θ, are one-to-one and the relationship [27] is as follows:

(6) $$f_{d}=\frac{2v\sin\theta }{\lambda }$$

which is also referred as a spatial–time relationship. This linear relationship between $f_{d}$ and θ can be represented in the spatial–time spectra, as Figure 2 shows.

In HRWS SAR imaging, the pulse repetition frequency (PRF), $f_{p}$, is typically significantly smaller than the instantaneous Doppler bandwidth. Hence, the Doppler spectrum is indeterminate, as depicted in Figure 2b, wherein the dots symbolize signal constituents originating from distinct directions within the same Doppler bin. Let us rephrase (4) in the following manner:

(7) $$S_{m}\left(f_{r},f_{d}\right)=\sum_{i=-N+1}^{N}s\left(f_{r},f_{d}+i\cdot f_{p}\right)\cdot \exp\left[-j2\pi \frac{x_{m}}{v}\left(f_{d}+i\cdot f_{p}\right)\right]$$

The 2N represents the Doppler ambiguity number, which should be less than the freedom degree of spatial M. The array steering matrix for the Doppler bin $f_{d}$ can be expressed as

(8) $$A\left(f_{d}\right)=\left[\begin{array}{ccccc} a_{-N+1}, & \cdot \cdot \cdot & a_{i}, & \cdot \cdot \cdot & a_{N} \end{array}\right]$$

The steering vector for the ith Doppler ambiguous component, $f_{d}+i\cdot f_{p}$, is

(9) $$a_{i}={\left[\begin{array}{ccccc} 1, & \cdot \cdot \cdot & \exp\left(-j2\pi \frac{x_{m}}{v}\left(f_{d}+i\cdot f_{p}\right)\right), & \cdot \cdot \cdot & \exp\left(-j2\pi \frac{x_{M}}{v}\left(f_{d}+i\cdot f_{p}\right)\right) \end{array}\right]}^{T}$$

where ${\left[•\right]}^{T}$ denotes the vector transpose operator. Then, the received array signal vector containing 2N Doppler ambiguous components can be given as follows:

(10) $$\begin{array}{l} S\left(r,f_{d}\right)=A\left(f_{d}\right)s\left(r,f_{d}\right)+e\left(r,f_{d}\right)\\ S\left(r,f_{d}\right)={\left[\begin{array}{ccccc} s_{1}\left(r,f_{d}\right), & \cdot \cdot \cdot & s_{m}\left(r,f_{d}\right), & \cdot \cdot \cdot & s_{M}\left(r,f_{d}\right) \end{array}\right]}^{T} \end{array}$$

where

(11) $$s\left(r,f_{d}\right)={\left[\begin{array}{ccccc} s\left(r,f_{d}+\left(-N+1\right)\cdot f_{p}\right), & \cdots & s\left(r,f_{d}+i\cdot f_{r}\right), & \cdots & s\left(r,f_{d}+N\cdot f_{p}\right) \end{array}\right]}^{T}$$

is the 2N unambiguous signal vector, and $e\left(r,f_{d}\right)$ is the additive white Gaussian noise vector, which is assumed to be spatial-independent and irrelevant to the signal. Furthermore, for convenience, we have replaced the time variable, τ, with the range variable, r, and $\tau =\frac{2r}{c}$. The current challenge is to expand the spatial–time spectrum and reconstitute the complete Doppler bandwidth spectrum.

### 2.2. Doppler Spectrum Reconstruction with Beamforming

This subsection introduces the basic principle of resolving Doppler ambiguity through spatial beamforming technology, specifically the fixed beamforming algorithm. This algorithm leverages the spatial–temporal spectrum relationship exhibited by stationary targets in SAR imaging. It separates and combines echoes from different azimuth angles within each Doppler bin of (7), ultimately producing a clear and unambiguous Doppler spectrum. The spatial filtering technique with beamforming for resolving Doppler ambiguity, assuming no phase error, is described using the following equations [28]:

(12) $$\hat{s}\left(r,f_{d}\right)=W^{H}\left(f_{d}\right)\cdot S\left(r,f_{d}\right)\approx W^{H}\left(f_{d}\right)A\left(f_{d}\right)s\left(r,f_{d}\right)$$

where $\hat{s}\left(r,f_{d}\right)$ represents the signal reconstructed by spatial filtering, and ${\left[\cdot \right]}^{H}$ denotes the conjugation operator; the spatial filtering for the 2N Doppler components is given as follows:

(13) $$\begin{array}{ll} W^{H}\left(f_{d}\right) & =H\cdot A^{-1}\left(f_{d}\right)\\ & ={\left[w\left(f_{d}+\left(-N+1\right)\cdot f_{p}\right),\cdots ,w\left(f_{d}+i\cdot f_{r}\right),w\left(f_{d}+N\cdot f_{p}\right)\right]}_{M\times 2N}^{H} \end{array}$$

where

(14) $$H=\mathrm{diag}{\left\{1,1,\cdots ,1\right\}}_{2N\times 2N}$$

and

(15) $$w\left(f_{d}\right)={\left[\begin{array}{ccccc} 1, & \cdot \cdot \cdot & \exp\left(-j2\pi \frac{x_{m}}{v}f_{d}\right), & \cdot \cdot \cdot & \exp\left(-j2\pi \frac{x_{M}}{v}f_{d}\right) \end{array}\right]}^{T}$$

is the spatial steering vector.

Once all the individual components of the spectrum have been retrieved, we reorganize them to create a clear and complete spectrum without any ambiguity. Subsequently, the traditional SAR imaging methods can be employed to concentrate the wide-swath image with exceptional precision. The mathematical model in (12) assumes an ideal condition with no channel phase error. However, channel imbalance is unavoidable, due to limitations in antenna machining accuracy, installation accuracy, and differences in receiver filter characteristics. Therefore, the ambiguous Doppler spectrum with channel phase error can be written as follows:

(16) $$S_{\varepsilon }\left(r,f_{d};\varepsilon_{r}\right)=\Lambda \left(\varepsilon_{r}\right)A\left(f_{d}\right)s\left(r,f_{d}\right)+e\left(r,f_{d}\right)$$

where $S_{\varepsilon }\left(r,f_{d};\varepsilon_{r}\right)$ is the multi-channel signal with channel phase errors, and the phase error matrix $\Lambda \left(\varepsilon_{r}\right)$ is

(17) $$\begin{array}{ll} \Lambda \left(\varepsilon_{r}\right) & =\mathrm{diag}\left[e^{j\varepsilon_{r}}\right]\\ & =\mathrm{diag}\left[e^{j\varepsilon_{r,1}},e^{j\varepsilon_{r,2}},\cdots ,e^{j\varepsilon_{r,M}}\right] \end{array}$$

The range-dependent channel phase error, $\varepsilon_{r,i}$, can be given by

(18) $$\varepsilon_{i}\left(r\right)=\kappa_{i}+\delta_{i}\cdot r$$

where the constant and first-order coefficient vectors are given as

(19) $$\kappa =\left[\kappa_{i}\right],\delta =\left[\delta_{i}\right],\left(i=1,2,\cdots ,M\right)$$

It is worth noticing that the channel phase error is usually a low-order function of slant range, so the linear approximation in Equation (18) is reasonable.

For clarity, the symbol $s_{\varepsilon }\left(r,f_{d};\varepsilon_{r}\right)$ will be replaced by $s_{\varepsilon }\left(r,f_{d};\kappa ,\delta \right)$ in the following deduction. The representation is in range-dependent form, where a first-order expression is used to achieve a compromise between model simplification and accuracy. In addition, extending polynomial modeling can be achieved simply by raising the order of Equation (2). Based on the range-dependent model, the phase error matrix, $\Lambda \left(\varepsilon_{r}\right)$, should be range-dependent with unknown parameters κ and δ, which are expected to be retrieved with the following sharpness maximization algorithm.

The construction of reconstructed Doppler spectra depends on the phase errors, which can be described using the following equation, by considering the range-dependent imbalance phase errors in the Doppler ambiguity resolving filtering with the non-adaptive filtering in (12).

(20) $$\begin{array}{l} \hat{s}\left(r,f_{d};\kappa ,\delta \right)=W^{H}\left(f_{d}\right)\cdot {\hat{\Lambda }}^{-1}\left(\varepsilon_{r}\right)\cdot s_{\varepsilon }\left(r,f_{d};\kappa ,\delta \right)\\ \;\;\approx W^{H}\left(f_{d}\right)\cdot {\hat{\Lambda }}^{-1}\left(\varepsilon_{r}\right)\cdot \Lambda \left(\varepsilon_{r}\right)\cdot A\left(f_{d}\right)s\left(r,f_{d}\right) \end{array}$$

where $\hat{\Lambda }\left(\varepsilon_{r}\right)=\mathrm{diag}\left[e^{j{\hat{\varepsilon }}_{r,i}}\right]$ denotes the estimated phase error matrix. It is evident from (14) to (17) that the presence of range-dependent imbalance phase errors causes the inversed-filtering method to fail in removing the Doppler ambiguity. The huge phase discrepancy between the ideal and actual steering vectors, which varies depending on the range, results in both signal loss and a substantial overlap of confusing components. As a result, the complete Doppler spectrum information becomes inaccessible. In the next section, the range-dependent phase calibration to estimate $\Lambda \left(\varepsilon_{r}\right)$ will be discussed in detail.

## 3. Range-Dependent Calibration with the Maximum Sharpness Optimization

Through the optimization of the pattern design of the SAR system, the Doppler spectrum becomes unambiguous, with a higher intensity focused around the Doppler centroid and a drop in intensity as one moves away from the centroid. When the channel phase error is present, the reconstructed azimuth Doppler spectrum appears flattened and spread out. This is because the Doppler ambiguous components are smeared, which leads to a decrease in sharpness. Hence, the channel phase errors that rely on the range will cause the Doppler spectrum rebuilt from the ambiguity reduction filtering to become wider. This inherent phenomena provides a direct method to obtain the phase error parameters by assessing the clarity of the reconstructed Doppler spectrum distribution. The initial definition of the reconstructed Doppler spectrum intensity is as follows:

(21) $$\begin{array}{l} I\left(r,f_{d};\kappa ,\delta \right)={\left|\hat{s}\left(r,f_{d};\kappa ,\delta \right)\right|}^{2}\\ \;\;\;\;=\hat{s}\left(r,f_{d};\kappa ,\delta \right)\cdot {\hat{s}}^{*}\left(r,f_{d};\kappa ,\delta \right) \end{array}$$

The sharpness function [21,22] is used to calculate the overall intensity of all reconstructed Doppler components in the Doppler spectrum. This function serves as a metric for the reconstructed Doppler spectra and is dependent on the phase error parameters.

(22) $$F\left(\kappa ,\delta \right)=\sum_{r,f_{d}}I{\left(r,f_{d};\kappa ,\delta \right)}^{2}$$

To maximize the sharpness of the reconstructed Doppler spectrum, one can take advantage of the sharpness optimization technique for phase calibration. The phase error parameters yield the highest level of sharpness optimization and is given by the following:

(23) $$\langle \hat{\kappa },\hat{\delta }\rangle =\arg\;\max\;F\left(\kappa ,\delta \right)$$

The sharpness optimization is generally convex within a local range, without any constraint. It is important to note that additional metric functions can be used to characterize the intensity distribution feature of the reconstructed Doppler spectrum. Examples include entropy, as described in [29], and contrast, as described in [22]. The primary rationale for choosing sharpness is mostly due to its convenience in deriving a simple yet effective gradient-based solver. The solution developed by Newton is a convenient and highly efficient method for obtaining an optimal estimation [21]. We implement an iterative gradient-based solver in the following content. We assume in the (k + 1)th iteration, the range-dependent phase error vector is updated with a gradient vector and Hessian matrix, which is given by the following equations:

(24) $$\hat{\kappa }\left(k+1\right)=\hat{\kappa }\left(k\right)+\Delta \kappa_{k}=\hat{\kappa }\left(k\right)-H^{-1}\left(\kappa \right)\cdot \frac{\partial F\left(\kappa ,\delta \right)}{\partial \kappa }$$

(25) $$\hat{\delta }\left(k+1\right)=\hat{\delta }\left(k\right)+\Delta \delta_{k}=\hat{\delta }\left(k\right)-H^{-1}\left(\delta \right)\cdot \frac{\partial F\left(\kappa ,\delta \right)}{\partial \delta }$$

where $H^{-1}\left(\cdot \right)$ denotes the Hessian inverse of the optimization function with respect to the parameter vectors. And the phase compensation process applies $\hat{\Lambda }\left(\varepsilon_{r}\right)$ on the multi-channel signal via ${\hat{\Lambda }}^{-1}\left(\varepsilon_{r}\right)\cdot s_{\varepsilon }$. We give the first order gradient derivations in the following equations.

(26) $$\frac{\partial F\left(\kappa ,\delta \right)}{\partial \kappa }=\sum_{r,f_{d}}2\cdot I\left(r,f_{d};\kappa ,\delta \right)\cdot \frac{\partial I\left(r,f_{d};\kappa ,\delta \right)}{\partial \kappa }$$

(27) ∂Ir,fd;κ,δ∂κ=∂s^r,fd;κ,δ2∂κ =2⋅Res^∗r,fd;κ,δ⋅∂s^r,fd;κ,δ∂κ

where

(28) $$\frac{\partial \hat{s}\left(r,f_{d};\kappa ,\delta \right)}{\partial \kappa }={\left[W^{H}\left(f_{d}\right)\cdot {\hat{\Lambda }}_{\varepsilon }^{-1}\cdot s_{\varepsilon }\left(r,f_{d};\kappa ,\delta \right)\right]}^{*}$$

and

(29) ∂F∂δ=∑r,fd4⋅I⋅Res^∗⋅∂s^∂δ =∑r,fd4⋅I⋅Res^∗⋅WHfd⋅Λ^ε−1⋅S˜c∗⋅r

The Hessian matrix of the sharpness optimization Hessian can be obtained using the following formulas. The component of Hessian, $H\left(\kappa \right)$, is deduced as follows:

(30) $$h\left(\kappa_{i},\kappa_{j}\right)=\frac{\partial^{2}F}{\partial \kappa_{i}\partial \kappa_{j}}=\sum_{r,f_{d}}2\cdot \left[\frac{\partial I}{\partial \kappa_{j}}\frac{\partial I}{\partial \kappa_{i}}+I\cdot \frac{\partial^{2}I}{\partial \kappa_{i}\partial \kappa_{j}}\right]$$

(31) ∂2I∂κi∂κj=∂2s^2∂κi∂κj=2⋅Re∂s^∂κi∗∂s^∂κi+s^*∂2s^∂κi∂κj

(32) $$\frac{\partial^{2}\hat{s}}{\partial \kappa_{i}\partial \kappa_{j}}=-W^{H}\left(f_{d}\right)\cdot {\hat{\Lambda }}_{\varepsilon }^{-1}\cdot {\tilde{s}}_{c}$$

And the second-order gradient component in the Hessian matrix, $H\left(\delta \right)$, is given by

(33) $$h\left(\delta_{i},\delta_{j}\right)=\frac{\partial^{2}F}{\partial \delta_{i}\partial \delta_{j}}=\sum_{r,f_{d}}2\cdot \left[\frac{\partial I}{\partial \delta_{j}}\frac{\partial I}{\partial \delta_{i}}+I\cdot \frac{\partial^{2}I}{\partial \delta_{i}\partial \delta_{j}}\right]$$

where

(34) ∂2I∂δi∂δj=∂2s^2∂δi∂δj=2⋅Re∂s^∂δi∗∂s^∂δi+s^*∂2s^∂δi∂δj⋅r2

(35) $$\frac{\partial^{2}\hat{s}}{\partial \delta_{i}\partial \delta_{j}}=-W^{H}\left(f_{d}\right)\cdot {\hat{\Lambda }}_{\varepsilon }^{-1}\cdot {\tilde{s}}_{c}$$

Typically, achieving the optimum level of sharpness optimization requires multiple iterations. The convergence can be regulated by specifying a maximum iteration number or by evaluating the discrepancy between two consecutive iterations. It is important to observe that, when addressing the optimization problem described in Equation (22), it is necessary to compute the first-order and second-order partial derivatives at each iteration. The calculation of the inverse of the Hessian matrix, which determines the search step, is performed for each iteration and requires a significant computational burden. To expedite the optimization of the phase error matrix, we provide an additional acceleration mechanism. By incorporating a termination condition, one can attain a harmonious equilibrium between the precision of the search step and the efficiency of the optimization process. The BFGS (Broyden, Fletcher, Goldfard, and Shanno) approach states that the Hessian matrix can be approximated using an updated formula. Thus, the Hessian and its inverse are initially determined through direct computation and, in subsequent iterations, the Hessian can be acquired through an updating process.

**Figure 3:**
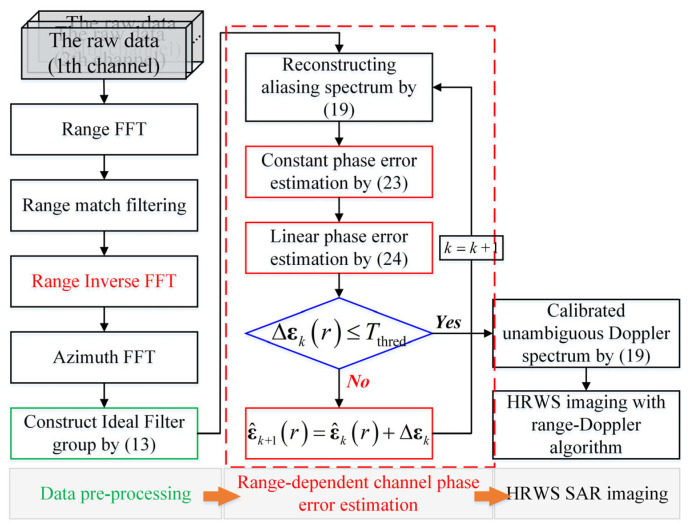
The flowchart of HRWS SAR imaging with range-dependent channel phase calibration.

Figure 3 displays the comprehensive calibration flowchart, which includes the suggested maximum sharpness optimization, for enhanced clarity. It is evident that the complete processing cycle consists of three distinct phases. The initial pre-processing stage involves applying range and azimuth Fast Fourier Transforms (FFTs) to the raw multi-channel data, transforming it into the two-dimensional frequency domain. The second stage involves calibrating the phase imbalance and reconstructing the complete Doppler spectrum without any uncertainty. Finally, SAR imaging processing using the traditional range-Doppler technique [30] is performed on the reconstructed entire Doppler spectrum to obtain high-resolution wide-swath (HRWS) imagery. Additionally, by utilizing the acquired phase errors to calibrate the raw data, one may effectively employ adaptive beamforming to resolve ambiguities. This approach leverages the adaptive beamformer, to enhance the performance of the resulting image. The rotation of beamformers is not the main focus of this study and, in the subsequent experiment, we conduct tests using the provided flowchart.

## 4. Experiments and Performance Analysis

The project involves the generation of azimuth multi-channel SAR data, utilizing the genuine space-borne SAR of Sentinel-1 provided in [3]. The original single channel SAR data are collected with a PRF of 1257 Hz, which is unambiguous in azimuth. To obtain 4-channel HRSW SAR data, we first up-sample the PRF from 1257 to 1676. Then, we performed data extraction along the slow time axis. As a result, the PRF of the multi-channel HRSW SAR data is now 419. This process simulates 4-channel uniform linear array data. Based on the given values, the distance between two adjacent channels in the array is approximately 8.427 m. In addition, a transmit bandwidth of 30 MHz yields a range resolution of 5 m. With 2048 range samples, this results in a range width of approximately 10.24 km. In our simulation experiments, we compare our proposed algorithm with the subspace projection algorithm [20] and the range-independent maximum spectrum sharpness (RIDMSS) algorithm from [21]. To conduct the comparison, we set the Doppler ambiguity number to three and the number of channels to four. Furthermore, we intentionally introduce random range-dependent phase errors to each channel in the original real-measured multi-channel SAR data, which creates a channel imbalance. Table 1 presents some parameters of the multi-channel SAR system.

**Figure 4:**
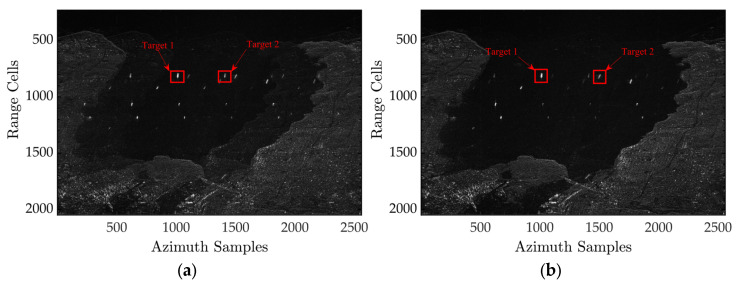

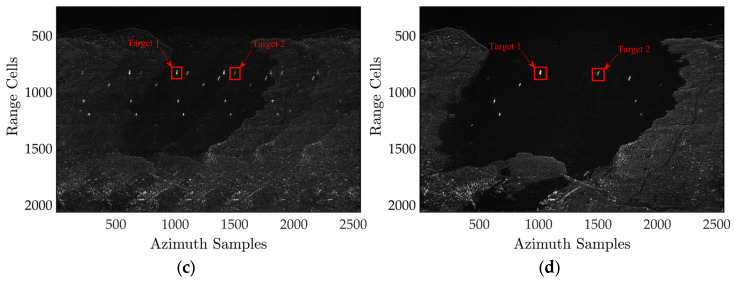
Imaging results with different channel error estimation algorithms. (**a**) Without compensation. (**b**) Result with the subspace projection calibration. (**c**) Result with the RIDMSS algorithm. (**d**) Result with the proposed algorithm.

The experiment presents the SAR imaging data obtained from both the subspace projection algorithm and our proposed approach, with and without phase calibration. These findings are shown in Figure 4a–d. Figure 4a clearly demonstrates that the imaging findings are significantly affected by the random phase errors between channels, resulting in noticeable ambiguities and blurring. The magnified sub-images clearly reveal the ambiguous components of ship targets, which would be considered as actual ships throughout the detection procedure. However, in the first experiment with a high signal-to-noise ratio (SNR), the findings from both calibrations, using multiple channels, effectively minimize ambiguity by employing the same spatial filtering technique. The inherent ambiguity components depicted in Figure 4a are effectively mitigated in the context of visual perception. This experiment illustrates that, in the presence of imbalanced phase errors, calibration processing is essential to attain an optimal level of ambiguity suppression performance.

As illustrated in Figure 4a, processing high-resolution wide-range SAR echoes directly in the presence of range-dependent channel errors cannot effectively reconstruct blur-free SAR images because the channel error violates the ideal spatial–time relationship, resulting in target ghosting. As shown in Figure 2b, the subspace-based approach can reduce the blurring component produced by constant channel errors. The remainder of the blurring energy generated by the range-dependent channel errors is, however, still clearly apparent. Furthermore, from Figure 4c, the range-independent based on the maximum spectrum sharpness algorithm converges to an incorrect extreme point after a finite number of iterations. Ignoring the linear component resulted in the algorithm mistakenly increasing the fuzzy component, while suppressing the actual echo energy, resulting in a significantly inferior algorithm performance. However, unlike the former ones, the proposed algorithm performs admirably. The ghosts of the reconstructed image are considerably suppressed by precisely estimating the constant and linear components in the range-dependent channel errors, resulting in a blur-free, high-resolution, and wide-swath SAR image.

**Figure 5:**
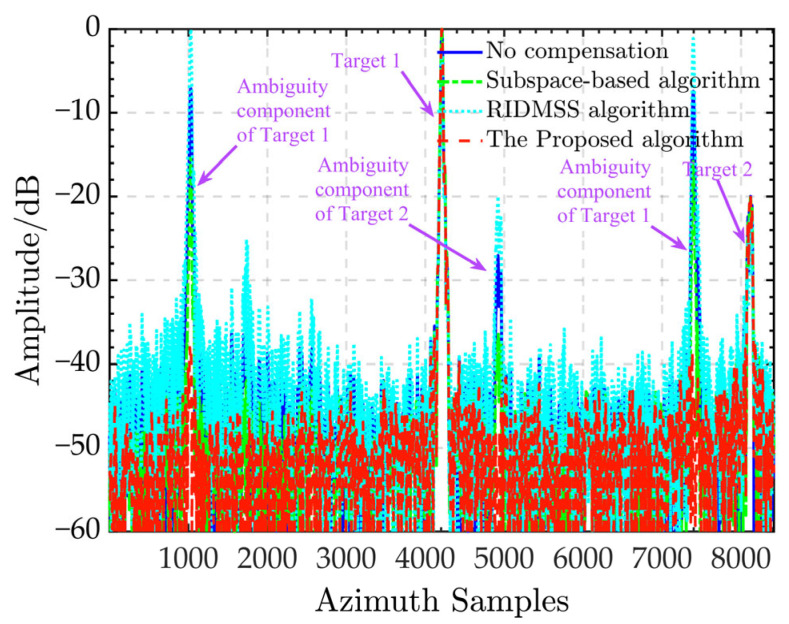
Azimuth profiles of ship targets from different calibrations.

Figure 5 depicts the azimuth profile to further demonstrate the ghost suppression performance of each algorithm in Figure 4. The subspace-based algorithm’s azimuth ambiguity-to-signal ratio (AASR) is roughly −16 dB, as seen in the figure. The AASR of the RIDMSS is around 2.2 dB due to the algorithm’s erroneous augmentation of the ambiguous component, while suppressing the genuine echo. Unlike the previous two, the suggested algorithm has an AASR of around −38 dB and efficiently removes the ambiguous components of both targets 1 and 2, indicating the effectiveness of the proposed approach.

**Figure 6:**
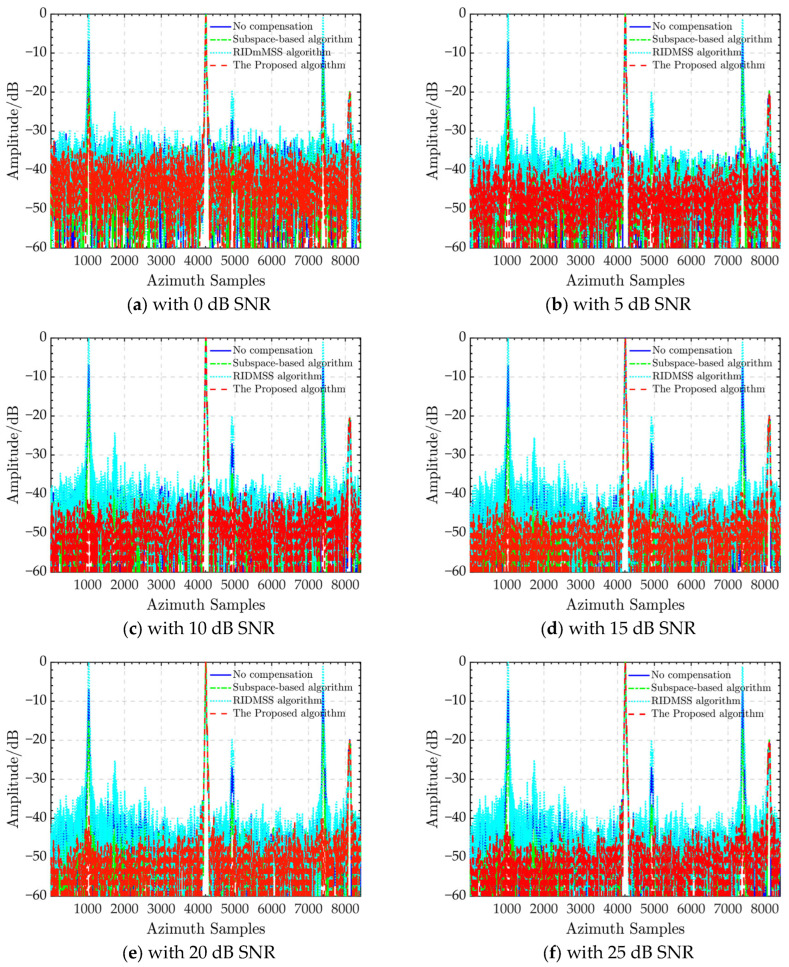

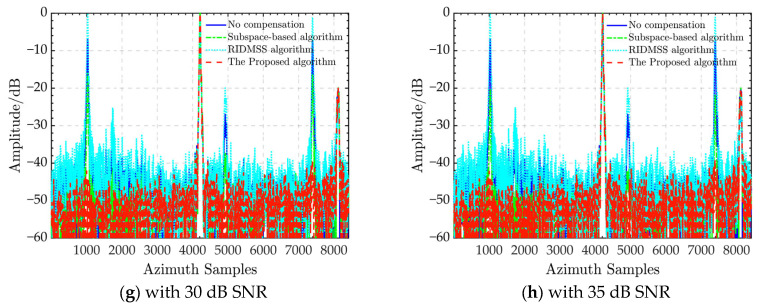
Azimuth ambiguity suppression of different calibrations under SNRs from 0 dB to 35 dB.

Furthermore, to demonstrate the robustness of the proposed algorithm, we used the MATLAB function *AGHWN* to add white Gaussian noise into the sentinel-1 radar data before pulse compression, resulting in different HRWS SAR data with signal-to-noise ratio (SNR) ranges ranging from 0 to 35 dB. We perform channel balance experiments with different SNRs using all algorithms based on this dataset. Their azimuth profiles are depicted in Figure 6. According to Figure 6a–h, the performance of the subspace-based approach improves marginally as the SNR increases, but the optimal AASR is never greater than −20 dB. The performance of the RIDMSS algorithm does not change significantly and the ghost energy remains comparable to the actual echo. Unlike the previous two algorithms, the suggested algorithm’s performance degrades slightly at low SNR, such as 0 and 5 dB, with an AASR of −22 dB and −27 dB, respectively. When the SNR increases, the AASR rapidly reaches −40 dB and remains steady. The suggested algorithm offers higher noise robustness than the previous two algorithms.

**Figure 7:**
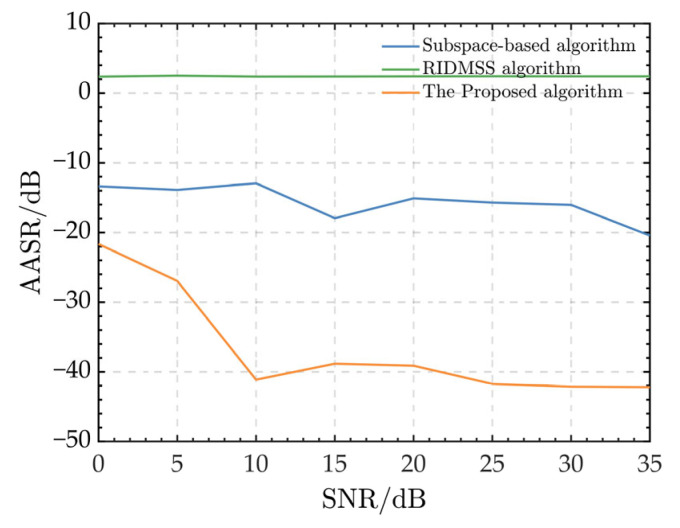
AASRs with different SNRs.

Figure 7 depicts the AASR curves of several algorithms with varying SNRs.

It is evident that the subspace-based algorithm achieves an AASR ranging from −10 dB to −20 dB. As the SNR increases, the AASR gradually improves. Furthermore, the proposed algorithm achieves better AASR performance compared to the subspace algorithm. The AASR quickly decreases from −22 dB to approximately −41 dB, as the SNR varies between 0 dB and 10 dB. When the SNR exceeds 10 dB, the AASR stabilizes around −40 dB. On the other hand, the RIDMSS algorithm exhibits the poorest AASR performance due to its inability to mitigate range-dependent errors. In summary, the AASR curves show that, in the presence of range-dependent channel phase errors, the suggested algorithm outperforms the other two algorithms in terms of robustness for HRWS SAR data.

## 5. Conclusions

The calibration of channel phase errors is a crucial step in achieving unambiguous SAR imaging with multi-channel SAR. This paper presents a phase calibration method that aims to enhance the sharpness of the reconstructed Doppler spectrum. By utilizing a gradient-based technique, the optimization problem for maximizing sharpness is solved, thereby reducing discrepancies in phase estimation. The proposed calibration approach provides precise and efficient phase correction performance, without requiring additional spatial freedom degrees. To validate the accuracy of the calibration, a real-data experiment is conducted, comparing it to a conventional approach and demonstrating the advantages of the proposed method. Finally, we believe that the proposed algorithm should be further improved in the future to correct both phase and amplitude. Moreover, it should be made adaptable to different spaceborne SAR imaging modes, as these features are currently absent from the algorithm.

## Figures and Tables

**Table 1:** System parameters.

Wavelength	0.056 m
Pulse width	41.750 µs
Sample rate	32.3 MHz
Bandwidth	30.1 MHz
Azimuth points	1920
Range points	2048
PRF	1257 Hz
Equivalent velocity	7062 m/s

## Data Availability

Data are contained within the article.
